# Deep Ocean Mineral Supplementation Enhances the Cerebral Hemodynamic Response during Exercise and Decreases Inflammation Postexercise in Men at Two Age Levels

**DOI:** 10.3389/fphys.2017.01016

**Published:** 2017-12-12

**Authors:** Ching-Yin Wei, Chung-Yu Chen, Yi-Hung Liao, Yung-Shen Tsai, Chih-Yang Huang, Rungchai Chaunchaiyakul, Matthew F. Higgins, Chia-Hua Kuo

**Affiliations:** ^1^Laboratory of Exercise Biochemistry, University of Taipei, Taipei, Taiwan; ^2^Department of Exercise and Health Science, National Taipei University of Nursing and Health Sciences, Taipei, Taiwan; ^3^Graduate Institute of Basic Medical Science, China Medical University, Taichung, Taiwan; ^4^Department of Health and Nutrition Biotechnology, Asia University, Taichung, Taiwan; ^5^College of Sports Science and Technology, Mahidol University, Salaya, Thailand; ^6^Department of Life Sciences, University of Derby, Derby, United Kingdom

**Keywords:** rehydration, minerals, trace elements, muscle power, inflammation, aging

## Abstract

**Background:** Previous studies have consistently shown that oral supplementation of deep ocean minerals (DOM) improves vascular function in animals and enhances muscle power output in exercising humans.

**Purpose:** To examine the effects of DOM supplementation on the cerebral hemodynamic response during physical exertion in young and middle-aged men.

**Design:** Double-blind placebo-controlled crossover studies were conducted in young (*N* = 12, aged 21.2 ± 0.4 years) and middle-aged men (*N* = 9, aged 46.8 ± 1.4 years). The counter-balanced trials of DOM and Placebo were separated by a 2-week washout period. DOM and Placebo were orally supplemented in drinks before, during, and after cycling exercise. DOM comprises desalinated minerals and trace elements from seawater collected ~618 m below the earth's surface.

**Methods:** Cerebral hemodynamic response (tissue hemoglobin) was measured during cycling at 75% VO_2max_ using near infrared spectroscopy (NIRS).

**Results:** Cycling time to exhaustion at 75% VO_2max_ and the associated plasma lactate response were similar between the Placebo and DOM trials for both age groups. In contrast, DOM significantly elevated cerebral hemoglobin levels in young men and, to a greater extent, in middle-aged men compared with Placebo. An increased neutrophil to lymphocyte ratio (NLR) was observed in middle-aged men, 2 h after exhaustive cycling, but was attenuated by DOM.

**Conclusion:** Our data suggest that minerals and trace elements from deep oceans possess great promise in developing supplements to increase the cerebral hemodynamic response against a physical challenge and during post-exercise recovery for middle-aged men.

## Introduction

A growing body of paleobiological evidence suggests that life on earth may have originated from the deep oceans (Gingerich et al., 2001; Kusky et al., 2001; Keller et al., 2017). If terrestrial organisms are evolved from deep oceans, sea-to-land migration could have compromised the nutritive complexity for all land survivors including descendants such as humans. In line with this concept, oral ingestion of components from deep oceans is likely to replenish any innate incomplete molecular complexity and increase the physical capacity of humans against entropic physical challenges. A proof-of-concept study has previously reported a substantially faster recovery (shortened from 48 to 4 h) on both leg muscle power (on a force plate) and aerobic fitness (maximal aerobic power on a cycle ergometer) in men stressed by an initial bout of exercise at high temperature (cycling at ~30°C until a 3% weight loss) with DOM supplementation (Hou et al., 2013). Similar results have been shown elsewhere, using different sources of mineral water from depths lower than 0.5 km below the earth's surface (Stasiule et al., 2014; Fan et al., 2016; Keen et al., 2016).

Another repeatable finding regarding the physiological benefits of DOM ingestion is its protective effect on vascular function in land animals (Miyamura et al., 2004; Radhakrishnan et al., 2009; Li et al., 2014). In contrast to surface ocean water containing a similar profile of major minerals (magnesium, potassium, calcium, sodium, and chloride), DOM demonstrated greater protective benefits against the development of atherosclerosis in rabbits fed a high cholesterol diet (Miyamura et al., 2004), suggesting that trace elements of the deep ocean water contributed to the attenuated vascular inflammation and improved vascular function. In surface ocean water where light is permeable (within ~200 m below the earth's surface), photosynthesis by marine organisms may exhaust biogenic components essential for optimal vascular functions (Miyamura et al., 2004).

The action of DOM on cerebral vascular regulation during exercise has not yet been documented. Cerebral blood supply increases during exercise as a result of increased brain metabolism (Querido and Sheel, 2007). However, vascular function deteriorates during aging (Barac and Panza, 2009). Maximal aerobic power declines from 40 years of age (Fleg et al., 2005). As the brain is the primary determinant of voluntary effort on muscle recruitment during exercise in humans (Kayser, 2003), cerebral hemodynamic function has been considered as a limiting factor for high-intensity performance (Subudhi et al., 2007; Rupp and Perrey, 2008). During a progressive maximal exercise to exhaustion on a cycling ergometer, cerebral oxygenation increases initially but decreased markedly shortly before exhaustion (Rupp and Perrey, 2008). Cerebral hemoglobin fluctuation, as an indicator of blood volume change in the frontal brain, can be monitored in real-time by near-infrared spectroscopy (NIRS) during cycling (Bay Nielsen et al., 2005). Based on the aforementioned reports of the effects of DOM on muscle power output and vascular function, we hypothesized that DOM supplementations can improve cerebral hemodynamic responses during and attenuate NLR response post high-intensity physical exertion in young and middle-aged men.

## Materials and methods

### Participants

This study recruited nine middle-aged men (aged 46.8 ± 1.4 year, body mass 81.4 ± 3.1 kg, height 175 ± 3 cm, BMI 26.5 ± 1.3, VO_2max_ 26.5 ± 1.3 mL/min/kg) and 12 young men (aged 21.2 ± 0.4 year, body mass 64.6 ± 1.6 kg, height 172 ± 1 cm, BMI 21.7 ± 0.5, VO_2max_ 45.2 ± 1.5 mL/min/kg) to determine cerebral hemodynamic and inflammatory responses during high-intensity cycling at 75% VO_2max_. Individuals with a history of musculoskeletal, orthopedic injury, or cardiovascular abnormality were excluded from taking part. The study was ethically approved by the University of Taipei Institution Review Board. All participants were asked to not ingest any alcohol or nutritional supplementation (such as caffeine-containing supplements) during the study including the washout period. Written inform consent was obtained from all participants after a detailed explanation of the study protocol.

### Experimental design

We conducted randomized placebo-controlled crossover trials in a counter-balanced order with a 2-week washout period using taste matched DOM and Placebo drinks. No measurement was conducted during the washout period. Only males were recruited for the study to avoid the influence of menstruation or potential acute anemia on brain hemodynamic measurement. DOM or Placebo was orally supplemented 12 h (600 mL bolus) and 1 h before exercise (1.8 mL per kg body mass (BM), every 15 min) during exercise (1.8 mL per kg BM at 15th min), and during post-exercise recovery (10 mL per kg BM in 2 h). All participants received the same meal (800–900 kcal) and water (600 mL bolus) the night before experimental trials and a standardized breakfast (200–250 kcal) 1 h before commencing exercise. The same meal was provided during the crossover trial. Participants had a repeatable dietary intake on testing days.

### Drinks

The desalinated DOM, taken from the West Pacific Ocean (618 m in depth), was provided by Pacific Deep Ocean Biotech (Taipei, Taiwan). DOM is defined by minerals and trace elements collected from ocean water 200 m below the earth's surface where sunlight is barely permeable. More than 70 minerals and trace elements existing in the ocean water have been documented (Farrington, 2000). DOM was filtered by a micro-filter (removal of microorganisms) and an ultra-filter (removal of any macromolecules and/or viruses) before use. Molecules sized above 1.5 KD were removed after this two-filtration procedure. To mask the taste difference between DOM and Placebo, the same amount of erythritol (3%) was added to each drink. Tap water purified by reverse osmosis process was used for making Placebo. The safety of long-term DOM supplementation has been tested and shows no adverse effect on survival rates in two different animal models (Liu et al., 2013; Liao et al., 2016).

### Exercise protocol

Maximal oxygen consumption (VO_2max_) and the associated workload (W_max_) were determined on a cycle ergometer (Monark, Sweden) at least 3 d before the start of experimental trials. The protocol for establishing VO_2max_ consisted of a 4-min warm-up, before beginning cycling at 100 W. The workload was increased incrementally by 25 W every 3 min until the participants could not continue to pedal despite constant verbal encouragement. The criteria used to establish VO_2max_ were a plateau of VO_2_ with increasing exercise intensity and a respiratory exchange ratio (RER) > 1.1 and an RPE score of 19/20. Expired gas was collected using a MetaMax 3B (Cortex Biophysik, Nonnenstrasse, Leipzig, Germany). Heart rate was measured by a Polar heart rate monitor (Lake Success, NY, USA). For experimental trials, participants cycled to volitional exhaustion at a constant work rate equivalent to 75% VO_2max_. Cerebral hemodynamic assessment was measured continuously during the first 20 min of exercise and time to exhaustion at 75% VO_2max_ was used as a measure of endurance performance. Trials were carried out at the same time of day (10:00 a.m.) to account for the influence of circadian rhythmic variation on exercise performance.

### Cerebral hemodynamic assessment

An optical probe of frequency domain multi-distance near-infrared spectroscope (NIRS) (ISS OxiplexTS, Champaign, IL, USA) was placed on the frontal brain and measured cerebral hemoglobin changes (detecting depth 2–2.5 cm) during the initial 20 min of exercise. Double-sided adhesive tape and an elastic band were used to secure the head probe in place. The NIRS oximeter was calibrated as per manufacturer guidelines prior to each test. All NIRS measurements (sampling rate: 1 Hz) were averaged over the last 60-s at each 5-min interval.

### Neutrophils-lymphocytes ratio (NLR)

NLR, a common marker of systemic inflammation, was measured before and 2 h after cycling at 75% VO_2max._ Venous blood samples were obtained for leukocyte analysis. The total numbers of leukocyte, neutrophils, monocytes, and lymphocytes were differentiated and quantitated using an automated hematology analyzer (Sysmex XT-2000, Sysmex Corp., Kobe, Japan) according to manufacturer's instructions.

### Lactate and glucose

Lactate and glucose were measured before and after 15th min during cycling at 75% VO_2max_. The fingerprick blood sample (10 microliters) was placed into a hemolyzing solution and serum was measured on a Biosen C-line glucose and lactate analyzer (EKF Diagnostic, Leipzig, Germany).

### Statistical analysis

Cycling time to exhaustion at 75% VO_2max_, total hemoglobin levels during exercise at the same time points and post-exercise NLR between two crossover trials were compared using a paired *t*-test. Data were also analyzed using a two-way analysis of variance (ANOVA) with repeated measures (supplementation and time effects) to determine main and/or interactive effects. Fisher's *post-hoc* test was used for pair comparison. A level of *P* < 0.05 was set for significance for all tests. Unless otherwise stated values are expressed as means ± SE. Performance data were also analyzed using an effect size with 95% confidence intervals (Watt et al., 2002).

## Results

The mineral and trace element profile of DOM is shown in Table 1. No difference in cycling time to exhaustion at 75% VO_2max_ was observed between Placebo and DOM trials (Placebo vs. DOM: 2558 ± 387 s vs. 2504 ± 446 s, respectively) in young men, but minimal increases in cycling time to exhaustion after DOM were noted in middle-aged men (Placebo vs. DOM: 5401 ± 855 s vs. 5601 ± 777 s, respectively, *P* = 0.08). This ~4% difference represents a minimal effect size of 0.09 (95% CI: −0.85 to 1.00) in endurance performance.

**Table 1:** Mineral and trace element profile of drinks.

**Mineral (mg/L)**	**Placebo**	**DOM**
Mg	0–15	146
Na	0–10	47
K	N.D.	46
Ca	0-15	0.2
**TRACE ELEMENT (**μ**G/L)**		
Li	N.D.	30
Rb	N.D.	10
B	N.D.	450

*N.D., not detectable; DOM. Deep ocean mineral*.

**Figure 1:**
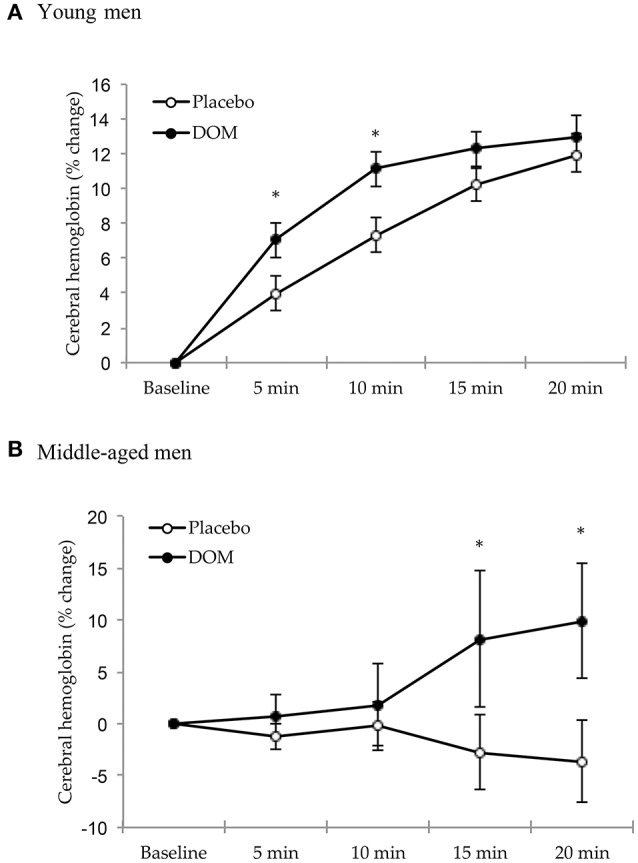
Cerebral hemodynamic response (tissue total hemoglobin) during cycling at 75% VO_2max_ for the young (aged 21.2 ± 0.4 years) **(A)** and middle-aged men **(B)** (aged 46.8 ± 1.4 years). According to two-way ANOVA with repeated measures, main effects of group and time are both significant (Group effect: *P* < 0.05; Time effect: *P* < 0.001) for young men. For middle-aged men, no change in cerebral hemodynamic response was observed at the same relative intensity (75% VO_2max_) during the Placebo trial, whereas significant increases in cerebral hemodynamic response during the DOM trial were observed after 15 min of cycling (*P* < 0.05). There was a significant group and time interaction (*P* < 0.01). * Significant difference against Placebo based on paired *t*-test, *P* < 0.05. DOM, Deep ocean minerals; Maximal oxygen consumption, VO_2max_.

The temporal changes of tissue hemoglobin levels in the frontal brain (detected by NIRS analysis) reflect the sensitivity of cerebral blood hemodynamic response (blood redistribution) against a physical challenge. Since the cycling time trials at 75% VO_2max_ showed a wide range of performance times amongst participants with the lowest cycling time around 21 min, we compared hemodynamic response data between both trials only for the first 20 min of cycling. For young men (Figure 1A), DOM supplementation enhanced the cerebral blood distribution by ~75% during the first 10 min of cycling compared to Placebo (paired-*t* test, *P* < 0.05). When data for the entire 20 min are included, the main effects of group and time are both significant (Group effect: *P* < 0.05; Time effect: *P* < 0.001) for young men. There was no interactive effect (2-way ANOVA, *P* = 0.297). For middle-aged men (Figure 1B), no change in cerebral hemodynamic response was observed at the same relative intensity (75% VO_2max_) during the Placebo trial, whereas significant increases in cerebral hemodynamic response during the DOM trial were observed after 15 min of cycling (paired *t*-test, *P* < 0.05). There was a significant group ^*^ time interaction (*P* < 0.01), but main effects of both group and time were not different when data for the entire 20 min are included (Group effect: *P* = 0.115; Time effect: *P* = 0.513).

**Figure 2:**
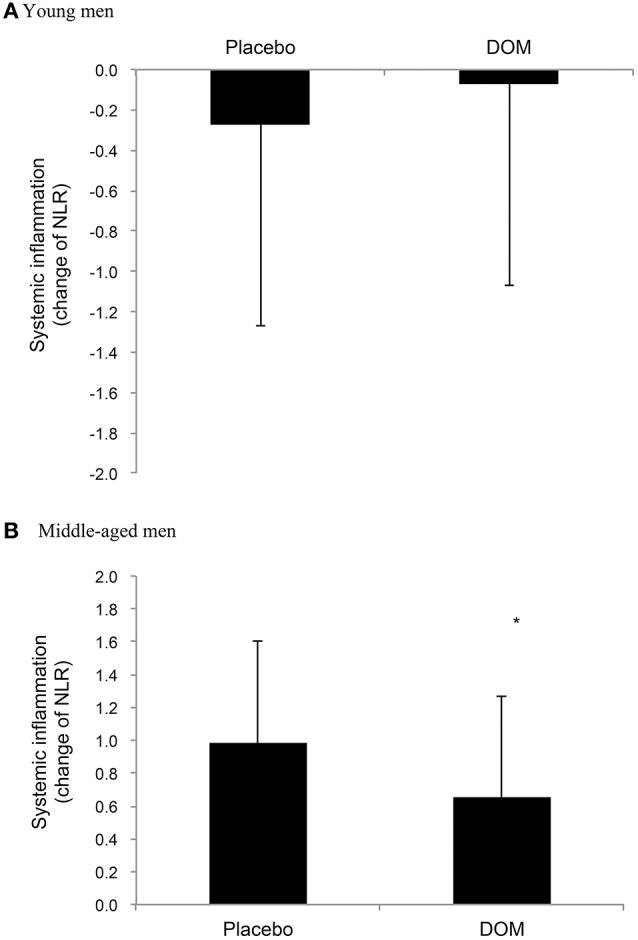
Neutrophil to lymphocyte ratio (NLR) change after exercise for the young (aged 21.2 ± 0.4 years) **(A)** and middle-aged men **(B)** (aged 46.8 ± 1.4 years). *Significant difference against Placebo, *P* < 0.05. DOM, Deep ocean minerals.

Table 2 shows similar heart rate, blood lactate, and glucose levels between the DOM and Placebo trials after the first 15 min of cycling at 75% VO_2max_ for young (a) and middle-aged (b) men, respectively. An increase in blood neutrophil to lymphocyte ratio (NLR) 2 h after cycling was only observed in middle-aged men. While no detectable difference between the DOM and Placebo trials was found in young men (Figure 2A), DOM supplementation attenuated the exercise-induced NLR response in middle-aged men by ~25% compared to Placebo (Figure 2B). Significant differences between the DOM and Placebo trials were noted when data are expressed as changes in NLR (paired *t*-test, *P* < 0.05) for middle-aged men, but not for young men.

**Table 2:** Physiological and metabolic responses of young **(A)** and middle-aged **(B)** men during high intensity cycling at 75% VO_2max_.

	**Treatment**	**Pre**	**15 min**	***P* (Interaction)**	***P* (Time)**	***P* (Treatment)**
**(A)**						
Heart rate (beats.min^−1^)	Placebo	90 ± 2	168 ± 4	0.54	<0.01	0.54
	DOM	90 ± 2	172 ± 3			
Lactate (mM)	Placebo	1.9 ± 0.1	9.8 ± 0.7	0.79	<0.01	0.99
	DOM	1.8 ± 0.1	9.7 ± 0.8			
Glucose (mM)	Placebo	4.6 ± 0.1	4.2 ± 0.2	0.51	<0.01	0.76
	DOM	4.8 ± 0.1	4.3 ± 0.1			
**(B)**						
Heart rate (beats.min^−1^)	Placebo	82 ± 4	134 ± 3	0.25	<0.01	0.25
	DOM	82 ± 4	131 ± 3			
Lactate (mM)	Placebo	1.9 ± 0.3	6.0 ± 0.3	0.55	<0.01	0.59
	DOM	1.5 ± 0.3	6.0 ± 0.4			
Glucose (mM)	Placebo	5.2 ± 0.1	5.4 ± 0.6	0.89	0.58	0.32
	DOM	5.1 ± 0.2	5.3 ± 0.4			

*P-value represents type 1 error of interactive and main effects of 2-way ANOVA. DOM, Deep ocean mineral water; Maximal oxygen consumption, VO_2max_*.

## Discussion

Vascular function is known to deteriorate with age (Barac and Panza, 2009), which may have ramifications on cerebral hemodynamic regulation during a physical challenge. Despite the protective effects of DOM on vascular function having been established with high reproducibility among animal studies (Miyamura et al., 2004; Radhakrishnan et al., 2009; Li et al., 2014), whether DOM can enhance cerebral hemodynamic responses during a physical challenge in men at various ages has not been previously documented. In this study, we found that mineral and trace elements from deep oceans can substantially increase the cerebral hemodynamic response during high-intensity cycling. The enhanced hemodynamic response with DOM was somewhat more pronounced in middle-aged men compared with young men at the same relative exercise intensity.

Improved cerebral hemodynamic response during high-intensity cycling provides mechanistic support to previous studies (Hou et al., 2013; Keen et al., 2016), which shows improved muscle power output in exercising men orally receiving DOM from more than 0.5 km below the earth's surface. As the brain is a critical determinant of muscle power output (Clark et al., 2014), the current findings suggest that components from deep oceans strengthen central command on muscle fiber recruitment mediated by accelerating blood supply to the frontal brain. However, high-intensity endurance performance was not significantly improved, suggesting that DOM had little influence on promoting fuel metabolism in contracting muscle.

A limitation of the present study is that mechanistic evidence to explain the role of specific minerals and trace elements from deep oceans for the enhanced cerebral hemodynamic response to exercise is not provided. We speculate that trace elements are the major components of the DOM enhanced cerebral hemodynamic response. DOM contains relatively higher amount of trace elements such as lithium and rubidium. Supplementation of lithium and rubidium are known to directly increase spontaneous motor activity levels in animals (Johnson, 1972). Additionally, manipulating lithium and rubidium concentrations affects the nervous system that controls movements in marine animals (Johnson, 1972; Hoffmann and Smith, 1979). To identify the key components of DOM, which modulate the cerebral hemodynamic response during exercise, can be a promising research area for improving quality of life for middle-aged men. Another limitation of the study is that the middle-aged participants in the study were also heavier in weight, compared with the young participants. Therefore, it is difficult for us to distinguish whether the observed differences between two age levels are due to age or weight.

Another novel finding of the present study is decreased systemic inflammation after exercise with DOM supplementation. The attenuated NLR increase suggests that DOM might either reduce the amount of damage or increase recovery rate after exercise. NLR is a commonly used marker of systemic inflammation and has been shown to be elevated 2 h following aerobic exercise, in an exercise volume dependent manner (Gabriel and Kindermann, 1997). The lowered systemic inflammation after exercise suggests that the increased molecular complexity provided by DOM supplementation improves the robustness of the cells against an entropic challenge.

The preventive effect of vascular inflammation by DOM has been previously reported (Li et al., 2014), suggesting that the lowered NLR found in the present study could be related to improved endothelial function. In rats, immunohistochemical staining data has shown that DOM supplementation decreases the proteins (MAP kinase signaling pathway) controlling cell proliferation and migration induced by vascular damage. Furthermore, DOM supplementation substantially delays the progression of atherosclerosis in animals (Miyamura et al., 2004; Radhakrishnan et al., 2009). However, we must acknowledge that these studies are involved with long-term DOM supplementation for 4–12 weeks in contrast to our study. Therefore, the underlying mechanism for the observed effects of DOM on cerebral hemodynamic responses warrants further investigation.

## Conclusions

The results of the present study strengthen the hypothesis that minerals and trace elements from deep oceans may increase nutritive complexity of humans against a physical challenge, supported by an enhanced cerebral hemodynamic response during cycling exercise and reduced systemic inflammation during recovery. Our findings suggest a promising application of using DOM to develop supplements for improving cerebral hemodynamic responses during physical challenges in middle-aged men.

## Author contributions

C-YW, C-YC, Y-HL, Y-ST, and C-HK conceived and designed the experiments; C-YW and Y-ST performed the experiments; C-YW and C-HK analyzed the data; C-YH, RC, MH, and C-HK wrote the paper.

### Conflict of interest statement

The authors declare that the research was conducted in the absence of any commercial or financial relationships that could be construed as a potential conflict of interest. The reviewer YS and handling Editor declared their shared affiliation.

## References

[B1] Barac A. Panza J. A. (2009). Mechanisms of decreased vascular function with aging. Hypertension 53, 900–902. 10.1161/HYPERTENSIONAHA.109.132308 19414643

[B2] Bay Nielsen H. Secher N. H. Clemmesen O. Ott P. (2005). Maintained cerebral and skeletal muscle oxygenation during maximal exercise in patients with liver cirrhosis. J. Hepatol. 43, 266–271. 10.1016/j.jhep.2005.02.039 15975685

[B3] Clark B. C. Mahato N. K. Nakazawa M. Law T. D. Thomas J. S. (2014). The power of the mind: the cortex as a critical determinant of muscle strength/weakness. J Neurophysiol. 112, 3219–3226. 10.1152/jn.00386.2014 25274345 PMC4269707

[B4] Fan H. Tan Z. Hua Y. Huang X. Gao Y. Wu Y.. (2016). Deep sea water improves exercise and inhibits oxidative stress in a physical fatigue mouse model. Biomed. Rep. 4, 751–757. 10.3892/br.2016.651 27284418 PMC4888009

[B5] Farrington J. W. (2000). Achievements in Chemical Oceanography. Washington, DC: The National Academics Press.

[B6] Fleg J. L. Morrell C. H. Bos A. G. Brant L. J. Talbot L. A. Wright J. G.. (2005). Accelerated longitudinal decline of aerobic capacity in healthy older adults. Circulation 112, 674–682. 10.1161/CIRCULATIONAHA.105.545459 16043637

[B7] Gabriel H. Kindermann W. (1997). The acute immune response to exercise: what does it mean? Int. J. Sports Med. 18, S28–S45. 9129261 10.1055/s-2007-972698

[B8] Gingerich P. D. Haq M. Zalmout I. S. (2001). Origin of whales from early artiodactyls: hands and feet of Eocene protocetidae from Pakistan. Science 293, 2239–2242. 10.1126/science.1063902 11567134

[B9] Hoffmann C. Smith D. F. (1979). Lithium and rubidium: effects on the rhythmic swimming movement of jellyfish. Cell Mol Life Sci. 35, 1177–1178. 10.1007/BF01963271 488270

[B10] Hou C. W. Tsai Y. S. Jean W. H. Chen C. Y. Ivy J. L. Huang C. Y.. (2013). Deep ocean mineral water accelerates recovery from physical fatigue. J. Int. Soc. Sports Nutr. 10:7. 10.1186/1550-2783-10-7 23402436 PMC3583772

[B11] Johnson F. N. (1972). Effects of alkali metal chlorides on activity in rats. Nature 238, 333–334. 10.1038/238333b0 4561839

[B12] Kayser B. (2003). Exercise starts and ends in the brain. Eur. J. Appl. Physiol. 90, 411–419. 10.1007/s00421-003-0902-7 12883902

[B13] Keen D. A. Constantopoulos E. Konhilas J. P. (2016). The impact of post-exercise hydration with deep-ocean mineral water on rehydration and exercise performance. J. Int. Soc. Sports Nutr. 13, 17. 10.1186/s12970-016-0129-8 27087798 PMC4833963

[B14] Keller M. A. Kampjut D. Harrison S. A. Ralser M. (2017). Sulfate radicals enable a non-enzymatic Krebs cycle precursor. Nat. Ecol. Evol. 1:0083 10.1038/s41559-017-0083 28584880 PMC5455955

[B15] Kusky T. M. Li J.-H. Tucker R. D. (2001). The Archean Dongwanzi ophiolite complex, North China Craton: 2.505-billion-year-old oceanic crust and mantle. Science 292, 1142–1145. 10.1126/science.1059426 11349144

[B16] Li P. C. Pan C. H. Sheu M. J. Wu C. C. Ma W. F. Wu C. H. (2014). Deep sea water prevents balloon angioplasty-induced hyperplasia through MMP-2: an *in vitro* and *in vivo* study. PLoS ONE 9:e96927. 10.1371/journal.pone.0096927 24824358 PMC4019650

[B17] Liao H. E. Shibu M. A. Kuo W. W. Pai P. Y. Ho T. J. Kuo C. H.. (2016). Deep sea minerals prolong life span of streptozotocin-induced diabetic rats by compensatory augmentation of the IGF-I-survival signaling and inhibition of apoptosis. Environ Toxicol. 31, 769–781. 10.1002/tox.22086 25727812

[B18] Liu H. Y. Liu M. C. Wang M. F. Chen W. H. Tsai C. Y. Wu K. H.. (2013). Potential osteoporosis recovery by deep sea water through bone regeneration in SAMP8 mice. Evid. Based Complement. Alternat. Med. 2013:11. 10.1186/1472-6882-13-11 24069046 PMC3773439

[B19] Miyamura M. Yoshioka S. Hamada A. (2004). Difference between deep seawater and surface seawater in the preventive effect of atherosclerosis. Biol. Pharm. Bull. 27, 1784–1787. 10.1248/bpb.27.1784 15516723

[B20] Querido J. S. Sheel A. W. (2007). Regulation of cerebral blood flow during exercise. Sports Med. 37, 765–782. 10.2165/00007256-200737090-00002 17722948

[B21] Radhakrishnan G. Yamamoto M. Maeda H. (2009). Intake of dissolved organic matter from deep seawater inhibits atherosclerosis progression. Biochem. Biophys. Res. Commun. 387, 25–30. 10.1016/j.bbrc.2009.06.073 19540194

[B22] Rupp T. Perrey S. (2008). Prefrontal cortex oxygenation and neuromuscular responses to exhaustive exercise. Eur. J. Appl. Physiol. 102, 153–163. 10.1007/s00421-007-0568-7 17882449

[B23] Stasiule L. Capkauskiene S. Vizbaraite D. Stasiulis A. (2014). Deep mineral water accelerates recovery after dehydrating aerobic exercise: a randomized, double-blind, placebo-controlled crossover study. J. Int. Soc. Sports Nutr. 11:34. 10.1186/1550-2783-11-34 25002835 PMC4083353

[B24] Subudhi A. W. Dimmen A. C. Roach R. C. (2007). Effects of acute hypoxia on cerebral and muscle oxygenation during incremental exercise. J. Appl. Physiol. 103, 177–183. 10.1152/japplphysiol.01460.2006 17431082

[B25] Watt K. Hopkins W. Snow R. (2002). Reliability of performance in repeated sprint cycling tests. J. Sci. Med. Sport 5, 354–361. 10.1016/S1440-2440(02)80024-X 12585619

